# Education Research: How Child Neurologists Counsel About Reproductive Health and Epilepsy: Gaps in Accuracy and Clarity in an Educational Needs Assessment

**DOI:** 10.1212/NE9.0000000000200261

**Published:** 2025-10-22

**Authors:** Laura Kirkpatrick, Erin Friel, Marie Clements, Christina Briscoe, Page B. Pennell, Traci M. Kazmerski, Jasmin Rivero-Guerra, Judy Chang

**Affiliations:** 1 Departments of Pediatrics and Neurology, University of Pittsburgh, PA;; 2 Department of Pediatrics, University of Pittsburgh, PA;; 3 Department of Pediatrics, University of Louisville, KY;; 4 Department of Neurology, Boston Children's Hospital, MA;; 5 Department of Neurology, University of Pittsburgh, PA; and; 6 Departments of Obstetrics, Gynecology & Reproductive Sciences and Internal Medicine, University of Pittsburgh, PA.

## Abstract

**Background and Objectives:**

The American Academy of Neurology (AAN) 2017 Women With Epilepsy Quality Measure advises all neurologists, including child neurologists, to counsel all 12- to 44-year-old female patients with epilepsy annually about at least 2 of 3 following topics: folic acid supplementation, interactions between antiseizure medications (ASMs) and contraception, and the effect of ASMs on pregnancy and/or fetal or child development. We hypothesized that child neurologists do not consistently conduct clear, factually accurate, and guideline-concordant counseling in this area. Therefore, as a targeted educational needs assessment, we performed a simulation-based evaluation of child neurologist knowledge and skill in this area to inform future educational intervention development.

**Methods:**

We recruited child neurology trainees (residents and fellows), attending physicians, and advanced practice providers (APPs) through a national listserv to engage in 3 virtual scenarios, simulating telemedicine appointments. We instructed them to perform reproductive health counseling for a female youth with epilepsy and their parent. We provided participants with detailed medical information about the patient before each scenario. We recorded and transcribed simulations. Coders performed content analysis to identify discussion of guideline-concordant counseling topics, assess factual accuracy, and analyze style of counseling for consistency with plain language.

**Results:**

Twenty-one individuals each performed the scenarios (11 attending physicians, 9 child neurology trainees, and 1 APP). Twelve (57%) addressed at least 2 of the 3 topics in the AAN quality measure across all scenarios. None (0%) performed guideline-concordant counseling without inaccuracies or omissions of key information across all scenarios. We identified no significant differences between attending physicians vs other provider types in stratified analyses. Qualitative analysis revealed that provider communication was rarely consistent with plain language standards. Common stylistic features included (1) long conversational turns, (2) multiple topics per turn, (3) complex sentence structures, and (4) use of jargon.

**Discussion:**

In case-based stimulation scenarios, most child neurologists discussed at least 2 of 3 AAN-recommended topics. However, participants commonly included factual inaccuracies and omitted key information, and did not use plain language, highlighting the need for training. Informed by our study findings, we will design and test a training intervention for child neurologists in this area as a future direction.

## Introduction

Adolescent and young adult female patients with epilepsy face distinct risks related to their reproductive health. Female patients with epilepsy face risks involving teratogenic and neurodevelopmental effects of many antiseizure medications (ASMs) and drug-drug interactions between ASMs and contraceptives.^1-3^ Some risks are specific to adolescence, including an elevated risk of unplanned pregnancy and a higher rate of teratogenic ASM prescription compared with older adults.^4-6^ The American Academy of Neurology (AAN) advises that neurologists, including child neurologists, begin to counsel female patients with epilepsy at least annually about epilepsy-specific reproductive health issues at age 12 years old.^7^ The 2017 Women With Epilepsy Quality Measure recommends provision of counseling on at least 2 of 3 reproductive health topics at least annually: the importance of daily folic acid supplementation to mitigate the neurodevelopmental risks of ASMs, the potential effects of ASMs and epilepsy on fetal or child development, and drug-drug interactions between ASMs and contraceptives.^7^

Previous research suggests that child neurologists commonly do not deliver guideline-concordant reproductive health counseling because of omitting it altogether, committing factual errors, or excluding important information.^8-14^ In prior mixed-methods research, child neurologists have reported low rates of performing counseling and have scored poorly on knowledge assessments in this area.^8-10^ Female patients with epilepsy have also reported receiving either no counseling or potentially inaccurate counseling.^11,12^ Yet, this previous research lacks sufficient granularity and details on child neurologist counseling performance to support development of training interventions to improve child neurologist knowledge and skill. Retrospective reports from child neurologists and patients lack examples of the exact content and wording of their counseling needed to understand strengths and weaknesses.

To better understand child neurologist counseling performance in greater detail to support educational intervention development, we conducted a simulation study in which we asked child neurologists to provide reproductive health counseling to an adolescent or young adult female with epilepsy.^15^ This simulation-based methodology allowed us to analyze the content and style of counseling to understand existing strengths and areas for improvement. This study reports results from this study related to guideline concordance, factual accuracy, and stylistic features of counseling. For this analysis, we hypothesized that less than 40% of participants would perform counseling addressing at least 2 of the 3 topics in the 2017 Women With Epilepsy Quality Measure without factual errors or omissions of key scenario-specific information across 3 scenarios.

A better, more detailed understanding of the content and accuracy of child neurologist attempts to provide reproductive health counseling will support future training development. Following Kern's 6-step process of curriculum development, this simulation study constituted a targeted needs assessment to inform creation of an educational intervention.^15^

## Methods

### Recruitment

We recruited eligible participants who were US clinicians in child neurology, including attending physicians, advanced practice providers (APPs), and residents and fellows (in postgraduate year 3 [PGY-3] or later in their training), as we plan to target our educational intervention to these groups. We conducted recruitment by email to the Child Neurology Society listserv. The email indicated that the purpose of this study would be to engage in simulated counseling about reproductive health and epilepsy for female youth. Individuals emailed the investigative team to express interest in the study. We did not request that participants refrain from reading about the topic before the study. We compensated participants for their time with a gift card. This study occurred from August through October 2024.

### Simulation Development

The principal investigator (L.K.) developed 3 scenarios simulating outpatient telemedicine visits. The scenarios were intended as an adaptation of ethnographic methods. The principal investigator aimed to vary (across the scenarios) the age and background of the patient, the epilepsy history of the patient including risk levels of their ASMs (in both teratogenicity and drug interactions with contraceptives), and whether a parent was present. The participants had access to detailed medical information about each simulated patient in advance of the simulation. The actors portraying the patient and/or parent had access to detailed character background including psychosocial history and sample dialogue for the actors portraying the patient and/or parent. A research team member who is a female youth with epilepsy (J.R.G.) reviewed the scenarios for face validity and proposed revisions. The scenarios were also reviewed for face validity by a senior coinvestigator with experience in communication science and reproductive health (J.C.). The research team engaged in a pilot session to test the feasibility and logistics of the scenarios before study initiation.

The final scenarios included (1) a 16-year-old girl with generalized epilepsy taking topiramate 200 mg/d and no folic acid supplement, accompanied by her mother; (2) a 19-year-old woman with focal epilepsy taking oxcarbazepine 1,200 mg/d and a folic acid supplement, presenting to the visit alone; and (3) a 15-year-old girl with juvenile absence epilepsy taking 1,250 mg of valproic acid per day and no folic acid supplement, accompanied by her mother. The scenarios are detailed in eAppendix 1.

### Simulation Procedures

The simulations occurred over Zoom teleconference software. We asked child neurologists to provide reproductive health counseling to a simulated patient portrayed by an actor in 3 scenarios. In 2 scenarios, an actor portraying the patient's parent was also present. We instructed participants to act as if they were in a telemedicine visit in-progress with an adolescent female patient with epilepsy and (when applicable) a parent. Participants engaged in the simulation from any environment in which they felt comfortable to proceed (i.e., home and office). All participants completed all 3 scenarios.

Before initiating the simulation, we introduced which team members would be playing which roles. The adolescent female patient with epilepsy was portrayed by a research team member who is a youth with epilepsy, currently an undergraduate student employed as a research assistant (J.R.G.). In the scenarios involving a parent, the parent was portrayed by another team member (E.F.), who is a female research coordinator. Both had received prior training in qualitative research including recorded interactions with research participants. The participants were aware that they were part of a study about counseling adolescents about reproductive health and epilepsy. The participants were aware that the actors were research team members, although no other information was reported about their background or motivation for conducting the research. There was no prior relationship between the team members and the research participants. No one else was present aside from the study participants and these team members.

We instructed the participant to perform their best attempt to conducting reproductive health counseling as they might during an outpatient visit, attempting to address important topics while being mindful of time limitations. We set a time limit of up to 15 minutes per scenario. We informed the participants that they could tell us when they were finished performing counseling to end the scenario. We informed participants that they could request that the parent leave the room to allow for confidential discussion with the patient and that if the parent agreed, the actor would turn their camera off. We provided the participant with written medical background information about the simulated patient and asked them to let the team know when they were ready to begin the simulation. When the participant expressed readiness, we began the simulation and started the timer. We audio-recorded the simulations and professionally transcribed them, with removal of identifying information before coding. There were no repeat sets of simulations for any participants. Following the simulations, we engaged in member checking with the participants through short debriefs and extended follow-up interviews about counseling quality. We did not take field notes during the simulations, debriefs, or extended follow-up interviews.

### Quantitative Outcomes

Our primary quantitative outcome was the proportion of the sample that performed counseling in each scenario that included at least 2 of the 3 topics listed in the 2017 AAN Quality Measure for Women With Epilepsy (i.e., folic acid supplementation, drug-drug interactions between ASMs and contraceptives, and the effects of ASMs on pregnancy and/or fetal or child development) without factual errors when discussing those topics and without major omissions of relevant important information based on the specific ASM used by the simulated patients. We prespecified key omissions for each scenario as part of the scenario development process. We defined key omissions for scenario 1, in which the patient is prescribed topiramate at 200 mg/d, as not mentioning the teratogenicity of topiramate and its potential for drug-drug interactions with some contraceptives. We defined key omissions for scenario 2, in which the patient is prescribed oxcarbazepine at 1,200 mg/d, as not mentioning the medication's potential for drug interactions with some contraceptives. We defined key omissions for scenario 3, in which the patient is prescribed valproic acid, as not mentioning the teratogenicity of valproic acid.

Secondary outcomes included identification of the most common errors made in key topic areas by the participants. An exploratory outcome was whether the proportion of participants who discussed at least 2 AAN-recommended topics without errors or key omissions varied between attending physicians vs other clinicians for each scenario.

### Quantitative Analysis

The principal investigator (L.K.), who is a board-certified pediatric epileptologist with clinical and research expertise in reproductive health and epilepsy, first coded the transcripts to indicate which topics were discussed and whether there were any key omissions in each scenario. A second coder, E.F., concurrently coded the transcripts, and both coders met on 3 occasions to reconcile their coding. Then, the principal investigator and 2 coinvestigators (M.C. and C.B.), who are board-certified pediatric epileptologists with a clinical and research interest in adolescent reproductive health and epilepsy, coded the transcripts to flag factually inaccurate information. At least 2 of the coders reviewed all transcripts for factual accuracy. The principal investigator and the other coders met on 2 occasions to review their coding and reconcile any discrepancies of interpretation of factual accuracy by consensus. The research team tabulated topics discussed, omissions, and factual errors for each scenario and across all scenarios.

For the exploratory outcome of differences in outcomes between attending physicians in child neurology and other clinicians, we performed a Fisher exact test of the proportion of the groups achieving the outcome of counseling on at least 2 AAN-recommended topics without factual inaccuracies or medication-specific omissions for each scenario, with an alpha level for statistical significance of 0.05. We conducted analyses in STATA version 18 (StataCorp, College Station, TX).

### Qualitative Outcomes and Analysis

Our qualitative analysis aimed to identify common stylistic features of child neurologist counseling, particularly whether counseling could be considered clear and comprehensible. We followed an approach previously published to analyze transcribed health care visits for features of verbal plain language.^16^ Owing to a lack of consensus standards for verbal plain language communication, prior investigators had adapted federal standards for written plain language, which have been widely used in both health care and non–health care settings, for identification of plain language features of verbal communication.^16,17^ Verbal plain language features, as adapted by these prior investigators^16^ from standards for written language, include avoidance of jargon, abbreviations, uncommon words, and unnecessary words; use of short sentences, short conversational turns, and 1 topic per turn; use of simple sentence structure; and use of potential examples or illustrations to help explain complex topics. Of note, in conversation analysis, a “turn” is “the words of [a participant] in a conversation before the next participant speaks.”^18^ For this study, the principal investigator (L.K.) developed a codebook informed by the prior investigators' codebook. Two team members (L.K. and E.F.) coded transcripts deductively using the codebook to identify the presence or absence of features of verbal plain language. We identified common stylistic themes based on the coding patterns. During the coding process, we evaluated for thematic saturation in an ongoing manner using published methods for determining saturation.^19^ We planned to perform at least 1 more simulation after achieving saturation for confirmation.

Two coders (L.K. and E.F.) inductively coded and identified themes from the debriefs. They met twice to reconcile coding.

We did not return transcripts of simulations to the participants for comment or correction. We performed qualitative analysis in Dedoose software (Sociocultural Research Consultants, Manhattan Beach, CA).

### Standard Protocol Approvals, Registrations, and Patient Consents

The University of Pittsburgh Institutional Review Board determined this study to be exempt, with no requirement for formal participant informed consent.

### Data Availability

Anonymized data not published within this article will be made available by request from any qualified investigator.

## Results

### Participant Demographics

Twenty-one clinicians in child neurology participated in the study, including 11 attending child neurology physicians, 8 child neurology residents (PGY-3 or above), 1 pediatric epilepsy fellow, and 1 APP. Six of the 11 attending physicians had completed an epilepsy fellowship. Of the remaining 5, 2 had completed neurodevelopmental disabilities fellowships and 3 were general child neurologists. No participants dropped out after initiating the simulations. The sample was 67% female (n = 14/21). The sample was 71% White/Non-Hispanic, 19% Asian (n = 4/21), and 10% multiracial (n = 2/21). One participant (5%) identified as Hispanic/Latino. Participants practiced in a variety of US Census regions, including the Northeast (38%, n = 8/21), the South (38%, n = 8/21), the West (19%, n = 4/21), and the Midwest (5%, n = 1/21). Among the attending physicians, there was a diversity of years in practice (median 9 years, interquartile range 6–15 years).

### Scenario Data

The recording failed for 1 participant in scenario 3. Thus, we have simulation data for 21 participants for scenarios 1 and 2, and 20 participants for scenario 3.

### Guideline-Concordant Counseling

No participants performed guideline concordant-counseling addressing at least 2 of the 3 AAN-recommended topics without factual errors or key omissions across all scenarios. Twelve participants (57%) addressed at least 2 of the 3 AAN-recommended topics across all 3 scenarios. One participant (5%) committed no factual errors across all scenarios. Six participants (29%) committed no key omissions of ASM-specific information across all scenarios. One participant (5%) stated factually inaccurate information in all 3 scenarios, 9 in 2 of the 3 scenarios (43%), and 10 in one of the 3 scenarios (48%). Figure displays participants' performance of guideline-concordant counseling across the 3 scenarios.

**Figure:**
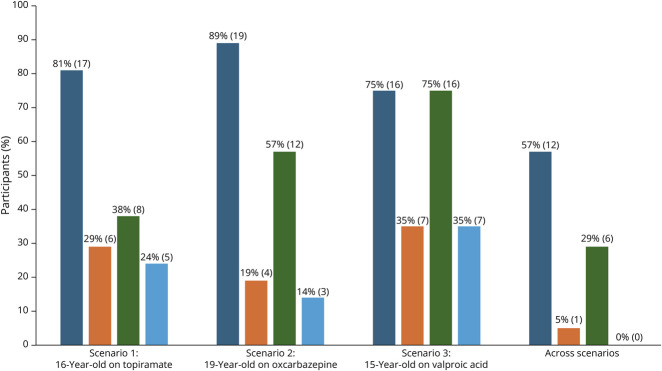
Provision of High-Quality Counseling by Scenario % (N) Dark blue—discussed at least 2 recommended topics. Orange—without factual errors. Green—without key omissions. Light blue—without factual errors or key omissions.

The 3 most common factual errors included (1) cautioning about the potential for valproic acid to reduce the efficacy of hormonal contraception at pregnancy prevention (i.e., an assumption that valproic acid has cytochrome P450 enzyme-inducing properties) (n = 5/20, 20%), (2) cautioning about the potential for hormonal contraception to reduce serum concentrations of ASMs (other than lamotrigine) in a clinically significant manner (n = 5/21, 24%), and (3) advising that topiramate is among relatively safer ASMs in pregnancy due to a perceived low risk of major congenital malformations and other complications (n = 5/21, 24%). Of note, all participants (n = 20/20, 100%) correctly disclosed the teratogenicity of valproic acid.

### Exploratory Analysis

For scenario 1, 3 of 11 (27%) attending child neurologists and 2 of 10 (20%) other clinicians achieved the outcome of discussing at least 2 AAN-recommended topics without factual inaccuracies or key omissions (*p* > 0.05). For scenario 2, 3 of 11 attending child neurologists (27%) and no other clinicians achieved this outcome (*p* > 0.05). For scenario 3, 5 of 11 attending physicians (45%) vs 2 of 9 other clinicians (22%) achieved this outcome (*p* > 0.05). There were no statistically significant differences in performance between attending child neurologists and other clinicians.

### Use of Plain Language

We achieved thematic saturation after coding the 20th set of participant simulations. We then performed 1 additional set of simulations to confirm saturation. Based on our qualitative analysis, participants rarely used plain language in counseling. Key themes included use of (1) long turns, (2) multiple topics per turn, (3) complex sentence structures, and (4) jargon.

#### Long Turns

When participants spoke, they often did so in a verbose didactic manner. All participants in all scenarios had at least 1 turn lasting at least 30 seconds long. An illustrative example of a long turn from participant 20, scenario 3 is included here

So, for all of our people that are able to have children—uh, as you reported, you had started your menstrual cycles a while ago—even if you're not thinking of having a family or being sexually active, we strongly recommend all of our people with epilepsy take folic acid. Um, and in this case, uh, d—I would have to ask my pharmacist if it's 1 mg or 2 mg, and there's a few reasons for this—and it's only once a day—'cause a lot of my young adults tell me they don't like to take too many meds, which I understand. So, uh, with the folic acid, there are some really good nur—uh, protective effects against—for the developing baby, specifically, valproic acid, or Depakote, can decrease your folic acid levels, which can be, uh—not great for the baby, if you were to get pregnant. And then folic—supplementing that would help against that depletion. Having low folic acids is associated with some pretty serious congenital brain and spine malformations for any developing baby, so we strongly recommend folic acid at least 1 mg/d. I'll have to double check if we would do a higher dose with valproic acid.

#### Multiple Topics Per Turn

Participants also addressed multiple topics in a single turn. Long turns often addressed multiple topics, and turns addressing multiple topics were often long, as can be seen in the following quotation from participant 3, scenario 1:

For those kids, um, that they're on medication such as topiramate, there is a small chance of teratogenicity. I know that's a big word, meaning that it causes problem for the little baby or the fetus to grow and have, um, normal development, so it's really important for us to measure that. The best thing that you can do for not getting pregnant is being celibate, but this is insane to ask a teenager, and I would never ask anyone that, so, um, we always ask for those kids who—'specially for your age, to, um—who are on this medication, to also, um, start, um, um, not—like, anti-pregnancy medication at the same time, but topiramate is not as often [teratogenic], so that would be something to consider, and always practice, um, safe sex, and also, uh, make sure to use condoms and be careful. So what do you think about all the information that-that I threw at you?

In 1 turn, the participant states topiramate is a teratogen, defines teratogenicity, discusses monitoring neurodevelopment, and advises celibacy; then retracts advising celibacy, advises contraception, and discusses the extent of teratogenicity caused by topiramate; and then advises condom use. Although these topics are thematically linked, these topics would be more ideally addressed in separate turns with intervening pauses and checks for patient/family comprehension and questions. The participant checks understanding only at the end of this series of topics, also implicitly acknowledging a communication problem (“So what do you think about all the information that I threw at you?”).

#### Complex Sentence Structures

Participants sentences often consisted of trains of multiple clauses (i.e., with multiple subjects and verbs). In addition to excessive clauses, this theme also encompasses codes adapted from prior investigators for “uses unnecessary redundant words” and “does not use short phrases.”^16^ For example, participant 8 in scenario 2 said:

Um, the other aspect to all of this is in case there is an unplanned pregnancy at some point, um, for any particular reason, or even a planned one, the goal, um, as your neurologist, as the seizure doctor, is going to be to ensure that the epilepsy does not come in the way of, um, a healthy mom and a healthy baby. Mm'kay?

#### Jargon

Participants often used overly technical language which could be considered jargon. Most jargon pertained to teratogenesis and birth defects, for instance, “The Depakote has a higher risk of teratogenicity compared to other seizure medications” (participant 4, scenario 3) and “[Folic acid] may help prevent some of the, um, congenital malformations” (participant 7, scenario 2). A minor theme was that some participants presented jargon with a brief definition or explanation, while many other participants did not.

### Qualitative Subgroup Analysis

When stratifying qualitative coding by participant type (i.e., attending physician vs resident/APP), there were no substantive differences in communication practices because all groups regularly used long turns, multiple topics per turn, complex sentences, and jargon.

### Debriefs

Participants reported discomfort performing reproductive health counseling because of not routinely engage in this activity in practice. Some reported that they infrequently encounter female adolescents with epilepsy. Others reported that they omit counseling because of personal discomfort, concern that the patient/caregiver might become uncomfortable, or time limitations with competing priorities. Participants reflected that they have poor knowledge of ASM teratogenicity and drug interactions between ASMs and contraceptives. Several participants indicated that they would use just-in-time resources to prepare for counseling. A few indicated that they had used resources during the simulations.

## Discussion

In this simulation-based study in which child neurology clinicians were asked to perform their best attempt to reproductive health counseling, we found that most addressed key topics listed in an AAN Quality Measure, but none did so without committing factual errors or omitting key ASM-specific information. In addition, communication often violated plain language standards through excessively long turns, multiple topics per turn, complex sentence structures (especially long sentences), and jargon (particularly related to birth defects and teratogenicity). There were no statistically significant differences or substantive qualitative differences between attending physicians and residents/fellows/APPs in performance of the counseling. As per Kern's 6-step process of curriculum development, this study was planned as a targeted needs assessment to inform future development of an educational intervention.^15^ This future intervention will train child neurologists in providing guideline-concordant, accurate, and comprehensible counseling about epilepsy and reproductive health to female youth with epilepsy.

Our findings build on previous reports from child neurologists that they are concerned about the quality of their counseling in this domain and reports from young women with epilepsy who described receiving either misinformation or potentially confusing information from child neurologists about epilepsy and reproductive health.^8-14^ These previous studies suggested that child neurologists may not be performing high-quality counseling but did not offer specific information about counseling quality or examples of how and why.

This study elucidates the gaps in knowledge and skill of child neurologists in this counseling. The findings from this study will inform development of an educational intervention to train child neurology clinicians to better counsel female youth with epilepsy about their reproductive health. The study findings support that this intervention must address both child neurologists' knowledge and communication skills. Although child neurologists were aware of the important content areas to address in counseling (as defined by the AAN), they were not able to address these areas with accurate information or a clear presentation of information.

The study findings also implicitly support a lack of appropriate and effective role modeling of good counseling given the lack of marked differences in performance between attending physicians with years in practice and trainees. Given that learning (as per Bandura Social Learning Theory) often occurs through observing, modeling, and imitating behaviors, the lack of senior role models in this area likely has substantial downstream effects on the workforce.^20,21^ To address this gap, the training intervention should provide positive opportunities for observation, modeling, and imitation of effective skills as an educational strategy. We plan therefore to develop an educational intervention comprising brief didactic information (potentially in an asynchronous format) followed by synchronous skills modeling and practice. Given similar performance across training levels, this training may be appropriate for both Graduate Medical Education and Continuing Medical Education.

This training could also focus on a priority area for many clinicians: time efficiency. In past studies, child neurologists have often opined that a leading barrier to their engagement in reproductive health counseling for patients with epilepsy is the limited time during office visits.^8,10^ The findings of this study reveal that many clinicians, when attempting this counseling, use didactic counseling with long turns, which makes the counseling potentially both less comprehensible to the patient and family, and longer in duration. Education on succinct, accurate communication of key messages may improve both clarity and efficiency of counseling.

In addition to areas for improvement in communication skills, participants also exhibited areas for improvement in knowledge. Contraception seemed to be a key area for improving child neurologist knowledge. The 2 most common factual errors among the sample pertained to drug-drug interactions between epilepsy and contraceptives, which should therefore be a key learning objective of the didactic component of the future training intervention.

The findings of this study attest to the importance of medical education as a dissemination and implementation mechanism for clinical practice guidelines. The 2017 Women With Epilepsy Quality Measure from the AAN,^7^ which frames this study, provided advisement roughly on counseling content, with some linkages to AAN resources for scientific knowledge in this area and no associated linkages to resources on needed skills and optimal style of counseling. To achieve successful translation into practice, such quality measures must ideally be accompanied by educational interventions that effectively train clinicians in practice on both the knowledge and skills required to implement the recommendations into patient care.

There are several limitations of this study. Simulated counseling does not necessarily accurately represent actual counseling that would occur in practice. Furthermore, awareness of being observed is a powerful motivator to exhibit model behavior (i.e., through the Hawthorne effect).^22,23^ However, clinicians participating in this study were asked to make their best attempt to counseling. Because participants were asked to perform their best attempt, the Hawthorne effect reinforces rather than detracts from the directions given in this study. The participant sample also specifically included clinicians who volunteered for a study on reproductive health and epilepsy and therefore may be biased toward those with a particular interest and potentially skill in this area compared with their peers. However, all of these factors should potentially bias the findings toward more optimal performance of counseling. That counseling quality was nevertheless poor in accuracy and clarity attests to the robustness of the findings.

Of note, it is also a limitation that research team members, rather than professional actors, portrayed the patient and the parent in the simulation, but we consider it a strength that the simulated patient was portrayed by a female youth with epilepsy. The small sample size, while permitting in-depth qualitative analysis, may also limit generalizability of the findings. Finally, for analysis of clarity of communication, we drew on a previously published adaptation of federal written plain language standards for verbal health care communication. There is a distinct need instead for a more tailored framework to analyze spoken plain language including formal definitions of features such as “long turns.”

In conclusion, our study demonstrates that child neurologists may not be prepared to perform high-quality counseling about reproductive health and epilepsy as per AAN quality measures in both content accuracy and clarity of communication. Our findings will inform subsequent development of a training intervention on style and content of counseling for child neurologists about adolescent reproductive health and epilepsy. Facilitated skills practice and role-modeling will be important in this training. This training will also emphasize an efficient counseling style to both optimize clarity and patient-centeredness as well as time utilization in office visits.

## Glossary

AAN: American Academy of Neurology
APP: advanced practice provider
ASM: antiseizure medication
PGY-3: postgraduate year 3
